# Supplementary material for: Extent and causes of the collapse in the registration of innovative medications in Lebanon: A mixed-methods analysis
Source: PLoS One. 2025 Dec 26;20(12):e0330585. doi: 10.1371/journal.pone.0330585 (PMC12742800; doi:10.1371/journal.pone.0330585)
Supplement: S1 File — (PDF) [file pone.0330585.s001.pdf]

S1 File

We are conducting a study to evaluate the delay in the registration of new/innovative medications in Lebanon between 2014 and 2024. Our preliminary findings indicate that this delay has been increasing ever since the 2019 crisis, further widening the gap between the global approval of new drugs and their local registration. Through this interview, we aim to explore the root causes, implications, and potential solutions to address this pressing issue from your perspectives and experiences.

1. To what extent do you think relevant stakeholders, including the MOPH, industry leaders, and healthcare professionals, are aware of this delay in innovative drug registration that occurred after the 2019 crisis in Lebanon?
2. What do you believe are the main reasons behind the delay in innovative drug registration in Lebanon after the 2019 crisis, and what was the rationale or intended impact of this decision?
3. Do you think that this unwritten policy (no new drug registration) relied on solid evidence? Please elaborate.
4. The Minister of Public Health has decided to delay drug registration in the country until the establishment of an HTA committee, which will be responsible for evaluating and overseeing the registration of new drugs. What is your opinion on this decision? *Probe on the intended benefit as well as the challenges and delays this has caused, what triggered this decision, etc.*
5. What steps should be taken to ensure the HTA committee effectively addresses drug registration delays? (i.e. feasibility and timeliness)
6. Before 2019, the MoPH registered innovative treatments without including them in its tender, leaving access to those with private insurance or other sources of coverage/funds. Which do you think is fairer: registering treatments without tender inclusion or not registering them at all if not included in the tender? Please explain why you think so.
7. In your opinion, what are the intermediate and long term outcomes/consequences of the delay in innovative drug registration (drug lag)? *Probe on access to innovative and potentially more effective treatments, drug smuggling, loss of medical tourism, changes in patients' health outcomes or satisfaction with available treatment options, equity, delayed access to care, effects on the pharmaceutical market etc.*
8. Was this ever discussed or considered before implementing this policy?
9. Based on our discussion, do you think that this decision (delay in innovative drug registration) should be reversed? If No, why not. If yes, what would be the most effective way to reverse this decision?

10. How effective has the collaboration been between the MoPH, industry leaders, and other stakeholders in addressing or managing the delay during the crisis? What could improve this coordination?
11. Is there anything else you would like to share, particularly regarding solutions or priorities to address the issue?
