# Supplementary material for: Extent and causes of the collapse in the registration of innovative medications in Lebanon: A mixed-methods analysis
Source: PLoS One. 2025 Dec 26;20(12):e0330585. doi: 10.1371/journal.pone.0330585 (PMC12742800; doi:10.1371/journal.pone.0330585)
Supplement: S1 Table — *For products approved in 2024 whose ATC is still pending approval. ✝Combination of remaining ATC who make a very small proportion of our sample. ‡Therapeutic subgroup under “A – Alimentary tract and metabolism”, but it’s often reported separately due to its importance. (DOCX) [file pone.0330585.s002.docx]

**S1 Table**

| **ATC** | **2014-2019** | | **2020-2024** | |
| --- | --- | --- | --- | --- |
|  | **FDA-approved** | **Registered by MOPH** | **FDA-approved** | **Registered by MOPH** |
| Alimentary tract and metabolism | 24 | 2 | 15 | 0 |
| Antiinfectives for systematic use | 36 | 11 | 15 | 1 |
| Antineoplastic and immunomodulating agents | 89 | 43 | 90 | 0 |
| Antiparasitic products, insecticides and repellents | 6 | 0 | 4 | 0 |
| Blood and blood forming organs | 14 | 3 | 4 | 0 |
| Cardiovascular system | 7 | 3 | 9 | 0 |
| Dermatologicals | 7 | 1 | 8 | 0 |
| Drugs used in diabetes ^‡^ | 8 | 6 | 4 | 0 |
| Genito-urinary system and sex hormones | 3 | 0 | 3 | 0 |
| Musculo-skeletal system | 7 | 1 | 7 | 0 |
| Nervous system | 29 | 5 | 21 | 0 |
| Ophthalmologicals | 5 | 1 | 5 | 1 |
| Respiratory system | 6 | 1 | 2 | 0 |
| Systemic hormonal preparations, excl. sex hormones and insulins | 5 | 0 | 7 | 0 |
| Pending* | 0 | 0 | 34 | 0 |
| Various^✝^ | 2 | 1 | 3 | 0 |
| **Total** | **248** | **78** | **231** | **2** |
