# Supplementary material for: Extent and causes of the collapse in the registration of innovative medications in Lebanon: A mixed-methods analysis
Source: PLoS One. 2025 Dec 26;20(12):e0330585. doi: 10.1371/journal.pone.0330585 (PMC12742800; doi:10.1371/journal.pone.0330585)
Supplement: S2 Table — *For products approved in 2024 whose ATC is still pending approval. ✝Combination of remaining ATC who make a very small proportion of our sample. ‡Therapeutic subgroup under “A – Alimentary tract and metabolism”, but it’s often reported separately due to its importance. (DOCX) [file pone.0330585.s003.docx]

**S2 Table**

| **ATC** | **2014-2019** | | **2020-2024** | |
| --- | --- | --- | --- | --- |
|  | **EMA-approved** | **Registered by MOPH** | **EMA-approved** | **Registered by MOPH** |
| Alimentary tract and metabolism | 19 | 2 | 13 | 0 |
| Antiinfectives for systematic use | 29 | 14 | 23 | 1 |
| Antineoplastic and immunomodulating agents | 80 | 45 | 88 | 4 |
| Antiparasitic products, insecticides and repellents | 0 | 0 | 1 | 0 |
| Blood and blood forming organs | 19 | 5 | 12 | 1 |
| Cardiovascular system | 7 | 4 | 7 | 1 |
| Dermatologicals | 1 | 0 | 6 | 1 |
| Drugs used in diabetes | 5 | 3 | 1 | 0 |
| Gentoo-urinary system and sex hormones | 2 | 0 | 3 | 0 |
| Musculo-skeletal system | 5 | 1 | 3 | 0 |
| Nervous system | 14 | 5 | 10 | 1 |
| Ophthalmologicals | 3 | 0 | 2 | 2 |
| Respiratory system | 7 | 2 | 2 | 0 |
| Systemic hormonal preparations, excl. sex hormones and insulins | 2 | 0 | 9 | 0 |
| Pending | 0 | 0 | 4 | 0 |
| Various | 1 | 1 | 4 | 0 |
| **Total** | **194** | **82** | **188** | **11** |
