# Supplementary material for: Extent and causes of the collapse in the registration of innovative medications in Lebanon: A mixed-methods analysis
Source: PLoS One. 2025 Dec 26;20(12):e0330585. doi: 10.1371/journal.pone.0330585 (PMC12742800; doi:10.1371/journal.pone.0330585)
Supplement: S3 Table — *The reason the lag time here is negative is because the MOPH had registered this medication based on prior approval by the other respective regulatory body. Meaning, Daclatasvir was approved by the EMA before the FDA, and its registration in Lebanon was based on the EMA’s approval. (DOCX) [file pone.0330585.s004.docx]

**S3 Table**

| **Regulator Comparison** | **Mean (Median) lag time in months** | **Greatest lag time in months** | **Shortest lag time in months*** |
| --- | --- | --- | --- |
| FDA versus MOPH | 19.4 (15) | 70 (Olaparib) | -4 (Daclatasvir) |
| EMA versus MOPH | 12.4 (11) | 70 (Olaparib) | -41 (Icosapent ethyl) |
