# Supplementary material for: Extent and causes of the collapse in the registration of innovative medications in Lebanon: A mixed-methods analysis
Source: PLoS One. 2025 Dec 26;20(12):e0330585. doi: 10.1371/journal.pone.0330585 (PMC12742800; doi:10.1371/journal.pone.0330585)
Supplement: Attachment — Submitted filename: Response to Reviewers.docx [file pone.0330585.s006.docx]

**Response to Reviewers**

We thank the academic editor and reviewers for taking the time to review our manuscript and for their suggestions which were carefully considered and incorporated into the revised manuscript. A point by point response to each comment is presented below:

| **Academic Editor’s Journal Requirements Comments** | |
| --- | --- |
| **Additional Requirements** | **Author’s Response** |
| 1. Please ensure that your manuscript meets PLOS ONE's style requirements, including those for file naming. | 1. All style requirements including: file format, length, font, headings, layout and spacing, etc. have been reviewed, no changes were made. |
| 2. Please provide additional details regarding participant consent. In the ethics statement in the Methods and online submission information, please ensure that you have specified (1) whether consent was informed and (2) what type you obtained (for instance, written or verbal, and if verbal, how it was documented and witnessed). If your study included minors, state whether you obtained consent from parents or guardians. If the need for consent was waived by the ethics committee, please include this information. | 2. Information regarding participant consent was moved from the sampling and recruitment section of the methodology to the ethics statement section (lines 121-125).  Written consent and the process of documentation was further elaborated on as per the Institutional Review Board’s approval. |
| 3. PLOS requires an ORCID iD for the corresponding author in Editorial Manager on papers submitted after December 6th, 2016. Please ensure that you have an ORCID iD and that it is validated in Editorial Manager. To do this, go to ‘Update my Information’ (in the upper left-hand corner of the main menu), and click on the Fetch/Validate link next to the ORCID field. This will take you to the ORCID site and allow you to create a new iD or authenticate a pre-existing iD in Editorial Manager. | 3. The corresponding author’s ORCID iD was updated on the editorial manager as kindly requested. |
| 4. If the reviewer comments include a recommendation to cite specific previously published works, please review and evaluate these publications to determine whether they are relevant and should be cited. There is no requirement to cite these works unless the editor has indicated otherwise. | 4. While the reviewer did not suggest specific references to include, we have strengthened the manuscript in response to all received comments by adding additional supporting references and ensuring that all citations adhere to the journal’s formatting requirements. |
| 5. Please review your reference list to ensure that it is complete and correct. If you have cited papers that have been retracted, please include the rationale for doing so in the manuscript text, or remove these references and replace them with relevant current references. Any changes to the reference list should be mentioned in the rebuttal letter that accompanies your revised manuscript. If you need to cite a retracted article, indicate the article’s retracted status in the References list and also include a citation and full reference for the retraction notice. | 5. We have reviewed the reference list and made the following corrections:   - The publication date and URL of reference 3 was updated - The URL of the following references was updated: 17, 24, 28, 30,34, and 56 |
| **Reviewers' comments (Reviewer 1)** | |
| **Methods** | |
| 1. My comment concerns section 2.3 Qualitative analysis addressing Objective. Details on sample size calculations were not given (why 15 participants were chosen? What is the rationale behind the choice of participants' number according to their background?). Other aspects should include a summary of study participant characteristics. | 1.In line with qualitative research principles, we adopted an iterative approach to data collection, determining the final sample size once data saturation was reached. Interviews were conducted gradually, with invitations sent on a weekly basis to around 2-3 participants at a time. Data collection was concluded when the research team agreed that no new topics or insights were emerging, indicating that saturation had been achieved. The final sample size was thus not decided on prior to initiating the study, but after data collection. This has now been clarified in lines 218-222, and a relevant reference from the literature has been added (reference 38).  The backgrounds and characteristics of participants are provided in the results section lines 314-317. The authors are cautious of not to provide too much information on participants to protect their confidentiality. A balanced representation between public and private stakeholders was needed to capture diverse perspectives. Additionally, this was intended to ensure the findings do not solely represent the views of the private sector (potentially influenced by specific interests in innovative drug registration) nor those of the public and academic sectors ( which may interpret the delay differently). This clarification has been added in lines 317-319. |
| **Discussion** | |
| 1. My other comment concerns the discussion; In my opinion this paper's second recommendation should come first. | 1. We agree with the reviewer’s comment and have revised the order of our recommendations to prioritize the immediate resumption of innovative medication registration (lines 482-488). In addition, we have clarified the role of HTA as a tool to inform reimbursement decision-making, independent of the registration phase. This is also in line with the second reviewer’s comments regarding these recommendations. |
| **Reviewers’ comments (Reviewer 2)** | |
| **Introduction** | |
| 1. The knowledge gap is not stated with enough precision. The paper should clearly articulate that no prior research has quantified Lebanon’s drug approval lag over time or explored the policy rationale behind registration collapse in a crisis context.  The conceptual framing of “drug approval lag” is sound but underutilized. The introduction could make stronger and clearer links between the main systemic drivers of such delays, like limited regulatory capacity, declining market attractiveness, and severe fiscal constraints and how these factors specifically apply to Lebanon’s current situation. Doing so would help justify why the study is important and relevant beyond simply describing what happened. | 1. We thank the reviewer for these important suggestions. The knowledge gap is now explicitly articulated in the final paragraph of the introduction (lines 100-103). The revised paragraph highlights the evidence gap related to Lebanon’s post-crisis drug approval lag, our interest resides in its policy rationale, and emphasizes the broader significance of the topic. The paragraph concludes with a clear statement of the study’s objectives.  The authors thank the reviewer for the valuable comment regarding the need to further elaborate on the drivers of regulatory delays. As noted in the Introduction (lines 53–57), we acknowledge the already well known causes of drug approval delays in other settings, but we also clarify that such delays are highly context-specific and shaped by the structure and priorities of individual health systems. In our context, delays were a result of an informal policy to halt the registration of innovative medications, primarily driven by financial challenges. If we were to elaborate on delays related to regulatory human capacity for instance, we could divert attention away from the primary cause of delay. We have added a section in the Discussion that presents evidence from the literature on regulatory delays in other countries and explain how these experiences were intensified as a result of the crises in Lebanon (lines 472-479). We hope that this addresses the reviewer’s comment in addition to comment # 2 under the discussion. We remain open to any further suggestions. |
| **Discussion** | |
| 1. Shift from description to analysis: the discussion too often repeats the results without moving into deeper interpretation. It should critically analyze why the collapse occurred: for example, how political decision-making, institutional weaknesses, fiscal constraints, and market withdrawal interacted to produce the observed outcome. | 1. We have amended the first two paragraphs of the discussion to not only restate the main findings of the paper, but to also critically analyze the observed drug approval lag and the interlinking factors behind it. (lines 440 – 452) |
| 2.Comparative and contextual depth: the section lacks sufficient comparative framing. Situating Lebanon’s experience alongside other crisis-affected or resource-constrained countries (LMICs or regional countries) would strengthen external validity and help clarify whether these dynamics are unique or part of broader patterns in drug regulatory behavior. | We have expanded the fourth paragraph of the Discussion to compare Lebanon’s experience with that of other LMICs (lines 472-479). The literature is however scarce when it comes to such evidence, which is why we could not compare our results to those of other MENA countries (as they do not exist). |
| 3. Evidence for consequences: Assertions about patient outcomes, economic losses, and declines in medical tourism are plausible but not directly supported by the study’s data. These should be reframed as hypotheses for future research. | We thank the reviewer for this accurate observation. The consequences mentioned were identified by stakeholders interviewed during the qualitative phase of our study and are based on their observations rather than secondary literature. We have already concluded the respective paragraph (lines 459-461) with the following statement: “These implications have not been measured in Lebanon yet, but the growing drug approval lag exacerbates all the aforementioned risks, highlighting the urgent need for corrective action.” We have further added the following sentence to the limitations paragraph of the discussion in order to call for future research: “ Future research is needed to further explore the relationship between the drug approval lag identified and the resulting negative consequences.” |
| 4. Actionable policy recommendations: Recommendations remain high-level and generic. They should be prioritized (e.g., immediate vs. structural reforms) and explicitly linked to the findings. For example, showing how separating registration from reimbursement could directly address the informal policy bottlenecks identified. | 4. The recommendations have been categorized into one immediate measure and two structural reforms. This directly addresses the first reviewer’s comment regarding the need to resume registration and separate it from purchasing functions within the MOPH as a primary policy outcome. In addition, the role of HTA in informing reimbursement decisions has been further clarified (lines 485-488) |
| 5. Integration of policy theory: The discussion would benefit from a stronger theoretical framework, drawing on health policy analysis or crisis governance literature. This would elevate the paper from a descriptive case study to a more analytical contribution with broader policy relevance. | 5. We thank the reviewer for this valuable comment. The authors have applied an interpretive framework to illustrate the rationale behind the delay in innovative medication registration, which was integrated into the qualitative findings. This framework has guided the paper’s analytical approach from the beginning.  However, we believe that incorporating a policy analysis framework to the discussion would increase the complexity of the manuscript and potentially distract the main message we intended to publish. The authors have incorporated references in the discussion on relevant policy frameworks that support evidence-based decision-making in Lebanon, as previously recommended in the literature, thereby strengthening the paper’s theoretical and policy relevance. |
| 6. Limitations: The treatment of censored data is a major methodological limitation; without appropriate analytical techniques (e.g., survival analysis), time-to-approval estimates may be incomplete or misleading. | 6. As the reviewer has accurately pointed out, censored data was a major challenge in our paper. We have attempted to apply survival analysis using Kaplan–Meier, however due to the small sample size and due to the short post crisis period, the results generated were also misleading, as they identified a shorter drug approval lag post crisis. Censored data has been mentioned as a limitation in both the methodology and limitations section of the discussion. |
| **Conclusion** | |
| The conclusion restates results clearly but should go further in highlighting the key takeaway: that the near halt in drug registration is a systemic issue rooted in institutional capacity, financial constraints, and policy design.  Policy recommendations should focus on two priorities: resuming registration independently of reimbursement decisions and strengthening regulatory governance to prevent future collapses.  Emphasizing how these lessons apply beyond Lebanon, particularly to other crisis-affected health systems would strengthen the paper’s overall contribution. | We fully agree with the reviewer’s comment and have addressed it by clearly stating our main takeaways in the conclusions of both the abstract and the manuscript (lines 524-528). |

We thank the editor and reviewers for all the suggested modifications which have improved the quality and understanding of our manuscript. We remain available if any further requests are required.
